# Efficacy of buprenorphine for management of surgical castration pain in piglets

**DOI:** 10.1186/s12917-018-1643-5

**Published:** 2018-10-23

**Authors:** Abbie V. Viscardi, Patricia V. Turner

**Affiliations:** Department of Pathobiology, University of Guelph, 50 Stone Road E, Guelph, ON N1G 2W1 Canada

**Keywords:** Animal welfare, Analgesia, Buprenorphine, Pain assessment, Piglet, Castration, Behavior, Piglet grimace scale

## Abstract

**Background:**

Surgical castration is a painful procedure, performed routinely on commercial pig farms to prevent boar taint and reduce aggression. The objectives of this study were to assess the efficacy of 0.04 mg/kg buprenorphine (BUP) in reducing pain in castrated piglets, using behavioral indicators and vocalization analysis. This study also sought to further validate the Piglet Grimace Scale (PGS) as a pain assessment tool.

A pilot study first assessed the safety of BUP or 0.2 mg/kg butorphanol administration to piglets (*n* = 4 per treatment). When no side effects were noted with BUP, ten litters of 5-day old piglets (*n* = 60 total, 15 per treatment group) were used, and randomly assigned to one of four possible treatments: BUP (castrated or uncastrated), saline, or sham. Treatments were administered as an intramuscular injection 20 min prior to surgical castration. Piglets were video recorded 1 h pre-procedure, post-castration for 8 h and for another hour, 24 h post-procedure. Behaviors were scored continuously for the first 15 min of each hour and 511 still-images of piglet faces were scored using the PGS. Vocalizations were recorded from each piglet at three points in the study: at initial handling, injection, and castration.

**Results:**

Butorphanol caused some piglets to become groggy and vomit and was not further evaluated. BUP-castrated piglets demonstrated significantly fewer pain behaviors and less facial grimacing compared to saline-treated pigs (*P* < 0.0001 and *P* = 0.0073, respectively). There was no difference between the pain behaviours displayed by BUP-castrated piglets compared to BUP-uncastrated and sham piglets (*P* = 0.9986 and *P* = 0.7484). There was also no difference in PGS score between BUP-castrated and BUP-uncastrated piglets (*P* = 0.9376). Piglets in the BUP-castrated group produced vocalizations of similar frequency, amplitude, power, and energy to saline-treated piglets.

**Conclusions:**

Buprenorphine was highly effective in alleviating castration-associated pain behaviors and facial grimacing in piglets, without causing any obvious side effects. Its administration did not reduce piglet vocalizations at the time of castration. The PGS corresponded well to piglet pain behaviors and has utility as a pain assessment tool.

## Background

Surgical castration is a procedure performed routinely on piglets in North America to prevent boar taint and minimize agonistic behaviors [1]. It causes acute pain in piglets, as evidenced by behavioral and physiologic alterations after castration, including rump scratching, body spasms, high-frequency vocalizations, and increased blood cortisol levels [2–4]. However, piglets are generally not provided analgesia or anesthesia for pain relief. Canada and countries in the EU have recognized this as a significant piglet welfare concern and have guidelines that now require analgesia administration [5, 6]. Nonsteroidal anti-inflammatory drugs (NSAIDs), such as meloxicam and ketoprofen, are currently recommended for use in piglets to manage pain, yet previous research found the label dose (0.4 mg/kg) of meloxicam, a high dose (1.0 mg/kg) of meloxicam, or 6.0 mg/kg ketoprofen to be ineffective at alleviating surgical castration pain in piglets [4, 7]. The analgesic capacity of an NSAID is limited by the degree of tissue trauma caused by the surgical castration procedure, as a significant mechanism underlying NSAID-induced pain mitigation is suppression of pro-inflammatory prostaglandin synthesis [8]. Opioids, such as buprenorphine and butorphanol, are more potent analgesic drugs, binding to μ, δ, and κ opioid receptors in the brain, spinal cord, and peripherally to suppress central pain signal transmission [9]. Butorphanol has been used in combination with various drugs, such as xylazine-ketamine, medetomidine, azaperone-detomidine-ketamine, and midazolam-ketamine, in pigs to prolong sedation [10–13]. Butorphanol alone was found to be ineffective at reducing pain behaviors of piglets castrated at 8 weeks-old [14]. Buprenorphine has demonstrated efficacy in reducing pain and lameness in pigs [15, 16]. The ability of butorphanol and buprenorphine to alleviate pain in 5-day-old piglets following castration has not been assessed.

The Piglet Grimace Scale (PGS) is a novel pain assessment tool that examines specific facial feature alterations in piglets in response to an acutely painful event [17]. Similar species-specific scales have been developed for mice, rats, rabbits, horses, sheep, and lambs [18–23]. These scales are of interest for their non-invasive nature and ability to rapidly detect pain [24]. For appropriate validation of these scales, they must correspond well to known indicators of pain, such as behavior.

The objectives of this study were first to determine the safety of buprenorphine and butorphanol administration to piglets, and then to assess their efficacy in reducing pain in castrated piglets, using behavioral indicators and vocalization analysis. We hypothesized that piglets receiving an opioid pre-castration would show a significant reduction in vocalizations and pain behaviors. This study also sought to further validate the PGS by comparing it against castration-related pain behaviors. The findings of this work will be important for appropriate analgesic recommendations to alleviate piglet pain post-castration, leading to improved animal welfare, a topic of increasing societal concern [25].

## Results

### Part I- opioid pilot study

#### Behavioral observations

Approximately 30 min post-injection, piglets administered butorphanol became groggy, unable to stand or walk, and two of the four animals vomited. They remained in the farrowing pen, and the observers ensured there was enough distance between them and the sow to eliminate their risk of being crushed. Butorphanol-treated piglets did not experience any severe side effects to the drug (e.g., respiratory depression), and they fully recovered approximately 1.5 h post-injection. There were no side effects noted with buprenorphine administration.

### Part II- buprenorphine definitive trial

#### Behavioral observations

**Fig. 1:**
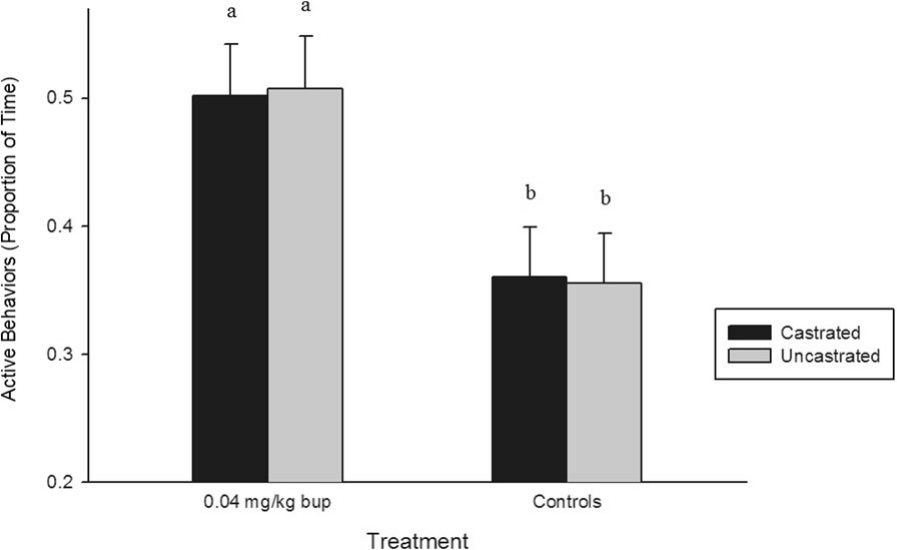
Mean proportion of time (± SE) piglets engaged in active behaviors in each treatment group. Different letters indicated significance

**Fig. 2:**
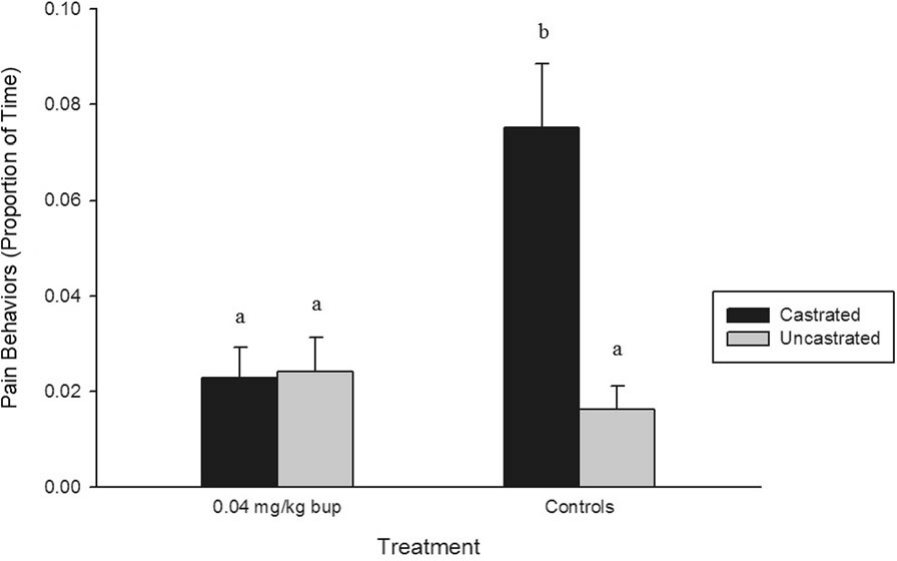
Mean proportion of time (± SE) piglets displayed pain behaviors in each treatment group. Different letters indicated significance

Four individual behaviors (lying: *P* < 0.0001, standing: *P* < 0.0001, tail wagging: *P* < 0.0001, and walking: *P* < 0.0001) and both grouped behaviors (active: *P* < 0.0001, and pain: *P* < 0.0001), were affected by treatment across all time points. Piglets in the BUP-castrated and BUP-uncastrated treatment groups spent significantly less time lying and more time standing, walking, and engaged in more active behaviours than piglets in the saline and sham treatment groups (*P* < 0.05) (Fig. 1). Saline-treated piglets wagged their tails and demonstrated significantly more pain behaviors than piglets in all other treatment groups (*P* < 0.05) (Fig. 2).

Eight individual behaviors and both grouped behaviors (active and pain) were significantly affected by time across the observation period: awake inactive (*P* < 0.0001), lying (*P* = 0.0016), sleeping (*P* < 0.0001), standing (*P* = 0.0004), suckling (*P* = 0.0288), tail wagging (*P* < 0.0001), walking (*P* < 0.0001), chewing (*P* = 0.0324), active (*P* = 0.0030), and pain (*P* < 0.0001). Regardless of treatment, at 0 h post-castration, piglets were significantly more active, spending more time standing and walking, and less time lying and sleeping compared to piglets at 4 h, 5 h, and 7 h post-procedure (*P* < 0.05). No rump scratching or trembling behavior was observed from any piglet pre-procedure and there were no significant behavioral differences between any of the treatment groups pre-castration (*P* > 0.05) (Table 1). Suckling and chewing behaviors were not significant after the Tukey-Kramer adjustment.

**Table 1:** Behavioral analysis of piglets (*n* = 60) pre-treatment and post-treatment across all litters and timepoints. Values presented are the proportional means ± SE

	Behavior^c^	Pre-Castration		Post-Castration						
		Treatment *P*-value	Pre-Treatment	Treatment P-value	Time P-value	Time*Treatment P-value	0.04 mg/kg BUP cast	0.04 mg/kg BUP uncast	Saline	Sham
Proportion (duration)	Awake inactive	0.9704	0.64 ± 0.05	0.6749	*<.0001*	0.2277	0.53 ± 0.02	0.50 ± 0.02	0.54 ± 0.03	0.52 ± 0.03
	Lying	–	0.53 ± 0.08	*<.0001*	*0.0001*	*0.0300*	0.49 ± 0.04^a^	0.44 ± 0.04^a^	0.68 ± 0.04^b^	0.67 ± 0.03^b^
	Nosing udder	0.0551	0.23 ± 0.05	*0.0248*	*0.0047*	0.9439	0.15 ± 0.03	0.17 ± 0.03	0.23 ± 0.04	0.25 ± 0.04
	Sleeping	0.6062	0.28 ± 0.06	*0.0187*	*<.0001*	0.7070	0.35 ± 0.07	0.32 ± 0.07	0.45 ± 0.07	0.49 ± 0.07
	Standing	0.7142	0.44 ± 0.08	*<.0001*	*<.0001*	*0.0228*	0.48 ± 0.08^a^	0.52 ± 0.07^a^	0.28 ± 0.06^b^	0.29 ± 0.06^b^
	Tail wagging	0.4929	0.02 ± 0.00	*<.0001*	0.4166	*0.0488*	0.00 ± 0.00^a^	0.00 ± 0.00^a^	0.07 ± 0.01^b^	0.01 ± 0.00^a^
	Walking	0.2945	0.16 ± 0.04	*<.0001*	*0.0001*	0.3728	0.23 ± 0.03^a^	0.25 ± 0.03^a^	0.07 ± 0.01^b^	0.09 ± 0.02^b^
	Active^d^	0.6394	0.47 ± 0.07	*<.0001*	*<.0001*	*0.0259*	0.51 ± 0.06^a^	0.56 ± 0.06^a^	0.33 ± 0.06^b^	0.33 ± 0.06^b^
	Pain^e^	0.2859	0.02 ± 0.00	*<.0001*	*0.0329*	*0.0002*	0.02 ± 0.00^a^	0.03 ± 0.00^a^	0.08 ± 0.01^b^	0.02 ± 0.00^a^

^a,b^Means with different superscripts in the same row differ significantly (*P* < 0.05); entries in italic font are statistically significant

^c^Only behavior variables that were significant post-treatment are presented

^d^Active behaviors include: nosing, suckling, walking, chewing, playing, running

^e^Pain behaviors include: stiffness, trembling, spasms, tail wagging and scratching

**Fig. 3:**
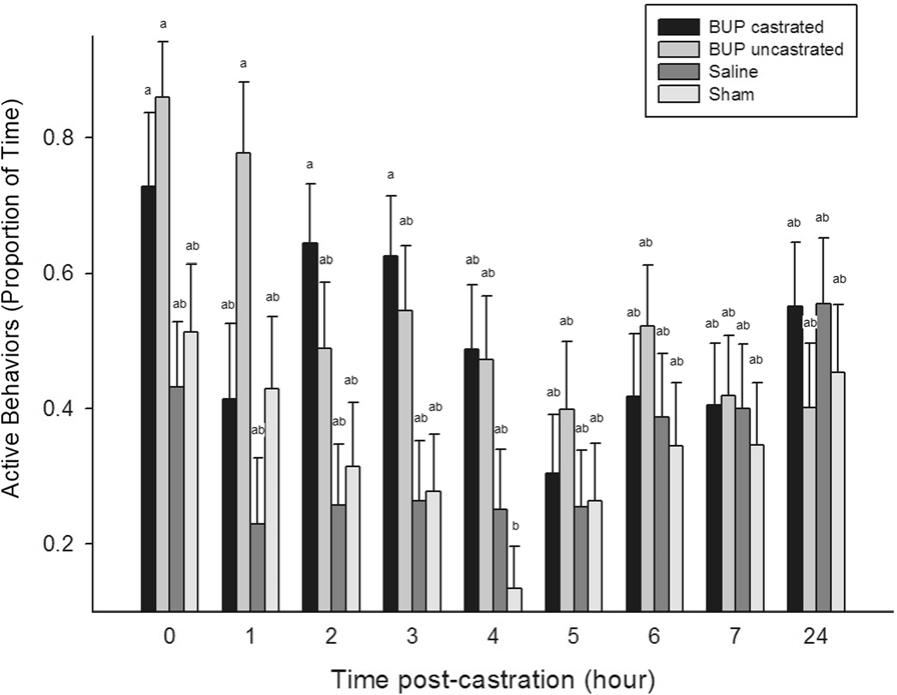
Mean proportion of time (± SE) piglets engaged in active behaviors within each treatment group across the observation period. Different letters indicated significance

**Fig. 4:**
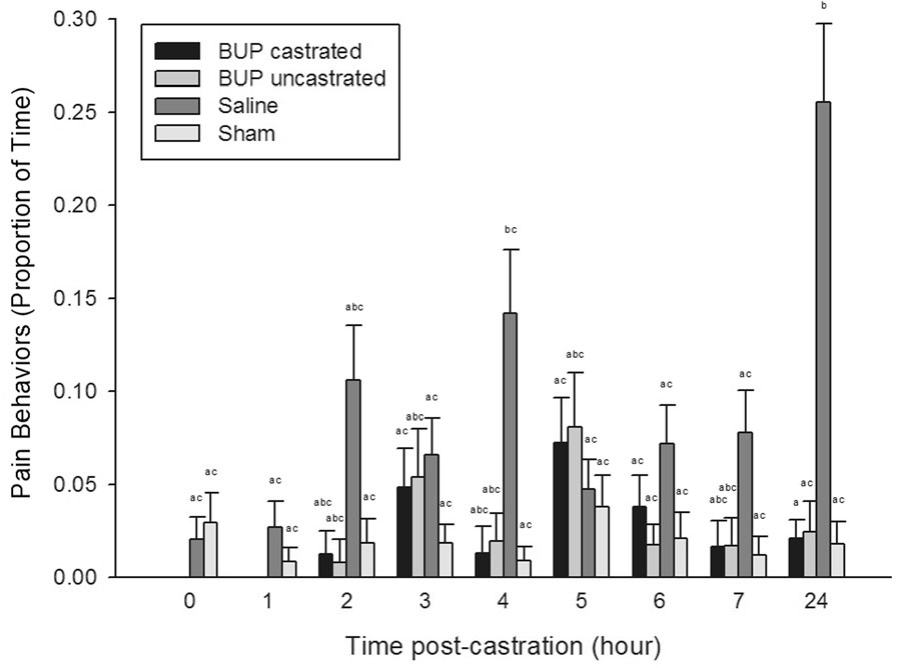
Mean proportion of time (± SE) piglets demonstrated pain behaviors within each treatment group across the observation period. Different letters indicated significance

A significant time x treatment effect was found for lying (*P* = 0.0300), standing (*P* = 0.0228), tail wagging (*P* = 0.0488), active (*P* = 0.0259), and pain (*P* = 0.0002). At 4 h post-castration, sham piglets were significantly less active, spending more time lying and less time standing than BUP-castrated piglets at 0 h, 2 h, and 3 h, and BUP-uncastrated piglets at 0 h and 1 h (*P* < 0.05) (Fig. 3). At 24 h post-castration, saline-treated piglets demonstrated significantly more tail wagging and pain behaviors than BUP-castrated piglets at 3 h, 5 h, 6 h, and 24 h, BUP-uncastrated piglets at 6 h and 24 h, and sham piglets at all post-castration time points (*P* < 0.05) (Fig. 4). Compared to themselves, saline-treated piglets displayed significantly more pain behaviors at 24 h than at 0 h, 1 h, 3 h, and 5 h–7 h post-castration.

#### Piglet grimace scale

**Fig. 5:**
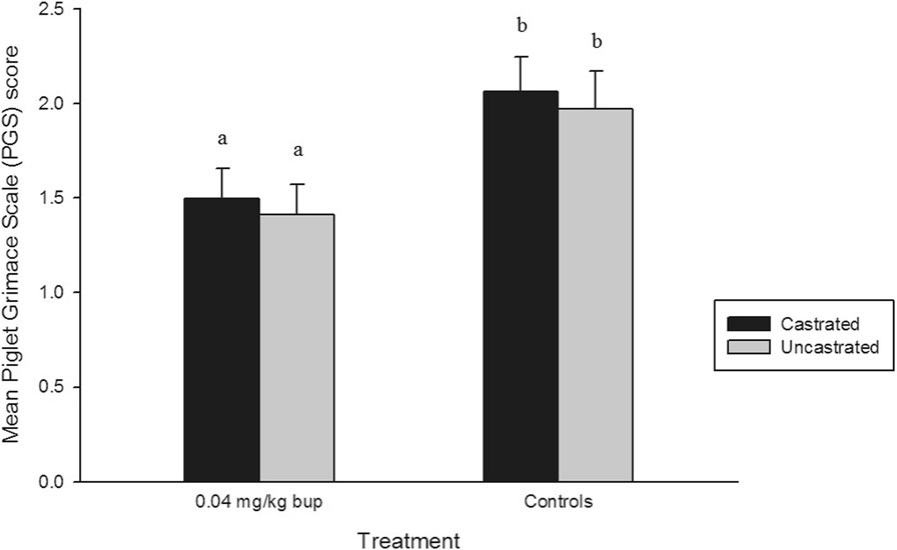
Mean Piglet Grimace Scale (PGS) scores (± SE) in each treatment group. Different letters indicate significance

There was a significant treatment effect on PGS score (*P* = 0.0003) (Fig. 5). BUP-castrated and BUP-uncastrated piglets grimaced significantly less than both saline (BUP-castrated: *P* = 0.007, and BUP-uncastrated: *P* = 0.001) and sham (BUP-castrated: *P* = 0.049, and BUP-uncastrated: *P* = 0.013) treatment groups. Across all time points, there was no significant difference in PGS score between BUP-castrated and BUP-uncastrated piglets (*P* = 0.944), nor was there a difference found between piglets in the saline and sham treatment groups (*P* = 0.974).

#### Vocalization

**Fig. 6:**
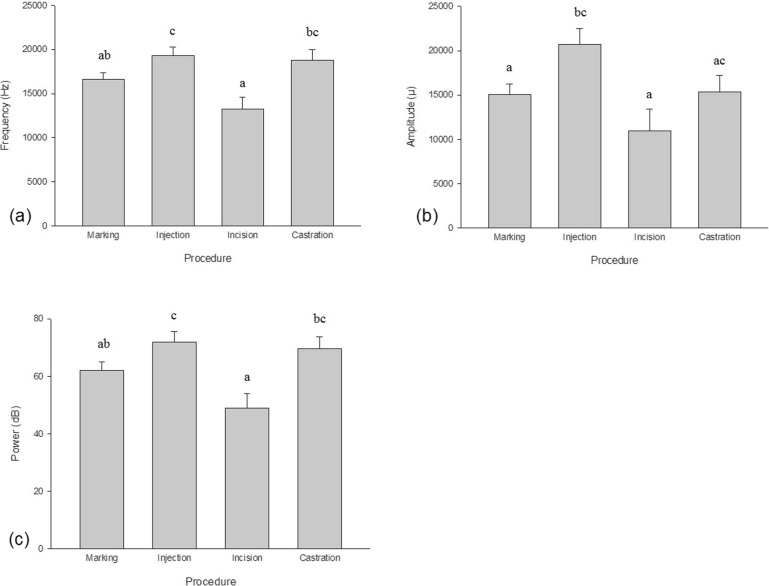
Vocalization (**a**) frequency, (**b**) amplitude, and (**c**) power (± SE) of all piglets undergoing each procedure. Different letters indicate significance

For both castrated-treatment groups, the scrotal incision produced piglet vocalizations that were significantly lower in frequency, amplitude, and power compared to the IM injection (*P* = 0.0003, *P* = 0.007, and *P* = 0.0001, respectively). When compared to castration (i.e., pulling and tearing of the spermatic cord to remove testicles), the scrotal incision produced piglet vocalizations significantly lower in frequency and power (*P* < 0.0001 for both). Injecting piglets resulted in vocalizations that were significantly higher in frequency, amplitude and power compared to marking piglets (*P* = 0.02, *P* = 0.04, and *P* = 0.02, respectively) (Fig. 6).

**Fig. 7:**
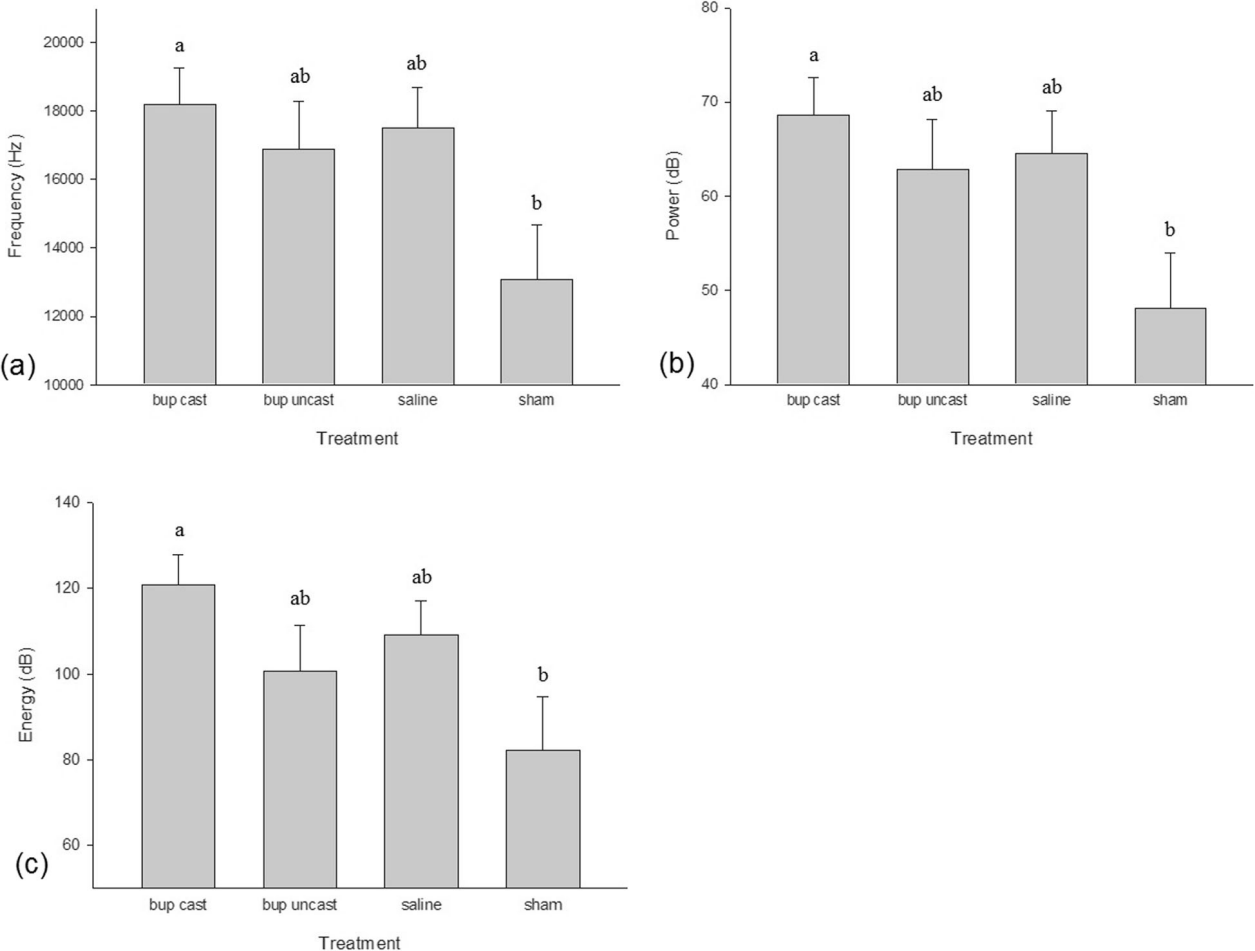
Vocalization (**a**) frequency, (**b**) power, and (**c**) energy (± SE) of piglets in each treatment group. Different letters indicate significance

Piglets in the BUP-castrated treatment group produced vocalizations significantly higher in frequency, power, and energy compared to piglets in the sham group (*P* = 0.04, *P* = 0.02, and *P* = 0.04, respectively) (Fig. 7). Buprenorphine administration did not reduce piglet vocalizations at the time of castration; piglets in the BUP-castrated group produced vocalizations of similar frequency, amplitude, power, and energy to saline-treated piglets (*P* = 1.00 for all).

#### Weight analysis

Sham piglets, with an average BW of 1.95 ± 0.14 kg, weighed significantly less than piglets in all other treatment groups (BUP-castrated: 2.48 ± 0.13 kg, *P* = 0.0044; BUP-uncastrated: 2.32 ± 0.12 kg, *P* = 0.045; saline: 2.44 ± 0.14 kg, *P* = 0.009).

## Discussion

This study examined buprenorphine efficacy in mitigating post-castration pain in piglets. Buprenorphine significantly reduced piglet pain behaviors such that no differences were observed between BUP- castrated and BUP- uncastrated or sham piglets at any post-castration time point (up to 24 h). Castrated and uncastrated buprenorphine-treated piglets were significantly more active than saline-treated pigs, showing no sedative effect with the 0.04 mg/kg dose. Animals often become less active but more restless when in pain [26]. Restlessness was difficult to assess with the ethogram used, but increased pain behavior and decreased activity was noted in saline-treated piglets in this study. Results from the treatment control group (i.e., piglets given buprenorphine but uncastrated) also verified no negative behavioral side effects are associated with buprenorphine administration. Saline-treated piglets wagged their tails significantly more than all other treatment groups. An increase in tail wagging after a painful event, such as castration or dehorning, has been observed in piglets, lambs, and calves [2, 7, 27–30], suggesting that this may be a useful pain indicator. Note that piglets in this study had not been tail docked and thus increases in tail wagging could not be attributed to tail stump hyperalgesia [31].

Increased piglet activity was observed immediately post-castration (at 0 h). This may be attributed to the stress of repeated handling, the IM injection and separation from the sow for 20–30 min. Piglets are also generally more active in the early morning and early evening [32]. At 24 h post-castration, saline-treated piglets demonstrated significantly more tail wagging and pain behaviors than any other treatment group and at most other time points. This increase in pain may be due to progression of the inflammatory process [33]. Buprenorphine-castrated piglets might have been expected to display more pain behaviors at 24 h post-castration, since the maximum duration of action of buprenorphine in swine is 12 h [34], but this was not observed. Future work should assess piglet pain beyond 24 h post-castration, to determine whether a single dose of buprenorphine provides sufficient post-castration analgesia for piglets.

After observing the significant sedative and emetic effects associated with butorphanol administration to piglets in the pilot study, this drug was not tested further. The sedative effects put piglets at greater risk of hypothermia or being crushed if they are immediately placed back in their pen, making it an inappropriate opioid for use in piglets. Buprenorphine was determined to be safe and there were no evident side effects in the pilot or definitive studies.

NSAIDs, such as meloxicam and ketoprofen, are licensed for use in swine and are the most practical drugs available for producers to administer to piglets for pain relief [35]. In terms of efficacy, both NSAIDs have had variable success in significantly reducing post-castration pain [4, 36]. A review of the existing literature by a group of swine experts determined the quality of evidence to support NSAID use was low, and they gave a weak recommendation for the use of NSAIDs to mitigate surgical castration pain [37]. It is clear that a more potent drug class, such as opioids, may be required for appropriate pain control in pigs, further evidenced by the results of this study.

Facial action unit analysis is an increasingly popular method to assess pain in animals, because it is non-invasive, quick, and easy to use [38]. To become a validated pain assessment tool, the PGS must be comparable to known indicators of pain. In this study, we compared PGS scores to the pain behaviors displayed by piglets. The observed pain behaviors corresponded perfectly to PGS results for buprenorphine-castrated, buprenorphine-uncastrated, and saline-castrated piglets (e.g., an increase in pain behavior corresponded to higher facial grimacing), and buprenorphine significantly reduced facial grimacing in castrated piglets. However, this was not noted for the sham-uncastrated group. Sham piglets were expected to demonstrate low facial grimacing, high activity, and low pain behaviors, as this group did not receive an IM injection and were not castrated. Instead, they demonstrated high facial grimacing, low activity, and low pain behaviors. The post-study weight analysis revealed that sham piglets as a group weighed significantly less on average than piglets in all other treatment groups. Low body weight (LBW) piglets have low survival rates through the first week of life [39], as they are at greater risk of crushing, starvation, and disease than piglets of average body weight [40, 41]. LBW piglets tend to miss more nursing bouts and spend more time alone [42]. It may be that the increase in facial grimacing and decrease in activity level of these piglets was due to weakness and discomfort, not pain. This is a confounder in this study and future work should ensure piglet weights are balanced across treatment groups.

Piglets emit distinct vocalizations associated with castration that have been attributed to pain [3, 43]. Buprenorphine did not reduce the frequency, amplitude, power, or energy of these vocalizations at the time of castration. Therapeutic concentrations of buprenorphine (0.1 ng/mL) are reached rapidly in pigs after IM injection (between 5 and 30 min) [32] and piglets were castrated 20 min after its administration. This suggests that buprenorphine alone does not provide sufficient analgesia to fully mitigate pain associated with surgical castration in conscious piglets. Previous studies have found that surgical castration-related stress vocalizations are reduced by CO_2_ anesthesia, a combination of ketamine-climazolam-azaperone anesthesia or intratesticular lidocaine injection [44–46]. However, these agents or combinations provide minimal post-operative analgesia and present greater limitations for on-farm use (e.g., sedated piglets can not be returned to the sow until after drug recovery).

The practicality of buprenorphine use on-farm currently is low. While it was highly effective, easy to administer, and one injection provided pain relief for at least 24 h post-procedure, it is a controlled substance that must be administered by a veterinarian and its use is currently prohibited (illegal) in pigs or other food-producing animals [47, 48]. The opioid abuse epidemic is another issue to consider. With more than 42,000 deaths in the U.S. related to opioid drug overdose in 2016, veterinarians have had to make major adjustments to the type of drugs they send home with clients and carry in their practice to reduce the risk of human abuse [49, 50]. Farmers appear to be disproportionately at risk, with three-quarters of U.S. farmers and farm workers having reported being directly affected by opioid dependence [51]. While the idea of widespread opioid use on-farm is inconceivable, identification of buprenorphine as a drug that significantly reduced surgical castration pain may encourage other researchers to focus on how to make this highly effective option for piglet pain management practical for use in swine production (e.g., through novel formulation or administration that is safe and limits the risk of abuse).

## Conclusions

Buprenorphine, when administered at 0.04 mg/kg IM, significantly reduced pain behaviors and facial grimacing in surgically castrated piglets for up to 24 h post-procedure, without evident adverse effects. It was not able to reduce vocalizations at the time of castration. The PGS corresponds well to the pain behaviors of piglets and has utility as a pain assessment tool. Future work should focus on potential solutions to the current limitations of using buprenorphine on-farm, as it is a highly effective analgesic agent that could improve the welfare of millions of piglets undergoing painful procedures each year.

## Methods

This study was conducted at Arkell Swine Research Station, an active research facility supported by the University of Guelph and the Ontario Ministry of Agriculture, Food and Rural Affairs (OMAFRA). All animal use and procedures were approved by the University of Guelph Animal Care Committee (Animal Utilization Protocol #3350). The institution is registered under the Animals for Research Act of Ontario and holds a Good Animal Practice certificate issued by the Canadian Council on Animal Care.

### Part I- opioid pilot study

#### Animals and treatments

A total of 8 Yorkshire-Landrace x Duroc male piglets (5-days-old, average BW = 2.19 ± 0.07 kg) from 2 different litters were used in this pilot study. Sows and piglets were housed in farrowing pens at the University of Guelph Arkell Swine Research Station (Arkell, ON, Canada). The floor space for each pen was 1.8 m × 2.4 m (6 ft. × 8 ft) and the farrowing crate was 0.8 m × 2.3 m (2.5 ft. × 7.5 ft). The farrowing rooms were maintained at ambient temperature (23 °C ± 0.5 °C) with lights on/off at 7:00 am/9:00 pm, and additional natural light was provided by windows in each room. Sows were fed ab lib 4 days after farrowing. The creep areas for piglets were heated to approximately 30–35 °C by means of a heat lamp.

Four piglets from each litter were used and randomly assigned one of two treatments: 0.04 mg/kg buprenorphine (Vetergesic 0.3 mg/mL; Champion Alstoe Animal Health Inc., Whitby, ON, Canada; extra-label use) or 0.2 mg/kg butorphanol (Torbugesic 10 mg/mL; Zoetis Inc., Kalamazoo, MI; extra-label use). Both drugs were administered intramuscularly (IM) and doses were derived from the literature [52]. Treatment groups were identified by a symbol (‘C’ or ‘D’) marked on the piglet’s back with a permanent marker prior to injection, to ensure that those involved in post-castration observations were blinded to treatment. A number was also marked on the back leg of each piglet for individual identification purposes.

#### Processing procedure

Piglets were weighed approximately 24 h prior to the study start for drug dose calculations, and then marked with a symbol and number. On the day of castration, male piglets were removed from their pen, placed in a transport cart, and treatments were administered. Approximately 20 mins later, piglets were surgically castrated by making one vertical incision over each testicle using a scalpel and tearing the spermatic cords. The piglets were then returned to their home pen. Castrations occurred between 8:00 am and 8:30 am and were all done by the same individual (AVV).

#### Behavior recording

Live observations were conducted for the first 1 h post-injection; if piglets responded negatively to the administered drug, they were quickly removed from their pen and assisted. One experienced observer blinded to treatment was placed outside each litter of piglets in this study and was instructed to note any unusual piglet behavior (e.g. grogginess, vomiting, distress, or lying isolated from littermates for an extended period). Video cameras (JVC GZ-E200 full HD Everio Camcorder, Yokohama, Japan) were also placed on tripods outside of the farrowing pens and piglets were video recorded during and after the live observations for 7 h. An individual not involved in the live observations assessed the video footage for behavioral signs of distress related to opioid administration.

### Part II- buprenorphine definitive study

#### Animals and treatments

A total of 60 Yorkshire-Landrace x Duroc male piglets (5-days-old, 1.07 to 3.34 kg BW) from 10 different litters were used in this study. Sows and piglets were housed in farrowing pens at the University of Guelph Arkell Swine Research Station.

Within each litter, piglets were randomly assigned to one of the following treatments: 0.04 mg/kg buprenorphine- castrated, 0.04 mg/kg buprenorphine- uncastrated, saline (castrated control), or sham (uncastrated control). Buprenorphine (BUP) was administered IM at 0.04 mg/kg (range: 0.2–0.5 mL/piglet). Saline was given IM at 0.2 mL/piglet. The sham treatment group was handled for approximately 30 s and did not receive an injection. Treatment groups were identified by a symbol (‘V’, ‘X’, circle or diamond) marked on each piglet’s forehead and back with a black permanent marker prior to castration. This was to ensure that the individual involved in post-castration observations and behavior scoring was blinded to animal treatment. A number was also marked on the back leg of each piglet for individual identification purposes.

#### Processing procedure

Piglets were processed as described for the pilot study. Castrations occurred between 8:00 am and 10:00 am. The sham treatment group were the only non-castrated piglets that underwent a simulated castration. All handling and technical procedures were conducted by female researchers to eliminate the potential confound of increased stress and an altered pain response in piglets exposed to male researchers, as reported in mice [53].

#### Behavior recording and scoring

Video cameras were placed on tripods outside of the farrowing pens and piglets were video recorded pre-procedure for 1 h. Immediately post-castration, piglets were video recorded continuously for 8 h, and 24 h post-procedure, piglets were recorded for 1 h (10 h of video data were collected in total for each pen of pigs). Videos were randomized using a random number generator (random.org) and the behavior of each piglet was scored continuously by one experienced observer for the first 15 mins of every hour of video collected using the Observer XT program (Version 12.0: Noldus Information Technology, Wageningen, The Netherlands) according to a detailed ethogram adapted from Hay et al. [2] (Table 2). The observer was blinded as to time point, litter, and piglet treatment; however, castrated piglets could be clearly distinguished from those that had not been castrated. A total of 9000 min (150 h) of behavior recordings were scored and analyzed.

**Table 2:** Ethogram used to score piglet behavior, grouped into feeding, locomotion, non-specific behaviors, pain-related behaviors, posture, and social cohesion (adapted from [2])

Behaviors	Description
Suckling	Teat in mouth and suckling movements
Nosing udder	Nose in contact with udder, up and down head movements
Playing	Springing, bouncy movements with littermates
Agonistic	Biting or fighting other littermates
Walking	Moving forward at a normal pace
Running	Trot or gallop
Awake inactive	No special activity, but awake
Sleeping	Lying down, eyes closed
Nosing	Snout in contact with a substrate
Chewing	Nibbling at littermates or substrates
Trembling	Shivering, as with cold
Spasms	Quick and involuntary contractions of the muscles
Scratching	Rubbing the rump against the floor, pen walls, or littermates
Tail wagging	Tail’s movements from side to side (or up and down)
Stiffness	Lying with extended and tensed legs
Lying	Body weight supported by side or belly
Sitting	Body weight supported by hindquarters and front legs
Standing	Body weight supported by four legs
Kneeling	Body weight supported by front carpal joints and hind legs
Isolated	Alone or with one littermate at most, distance of 40 cm separates the animal(s) from the closest group of littermates
Desynchronized	Activity different from that of most littermates (at least 75%)

Piglet behaviors were analyzed individually and then grouped into active, inactive and pain categories, to assess the piglet’s activity level throughout the observation period and the total amount of pain behaviors displayed [7]. Active behaviors included walking, running, playing, nosing, chewing, and suckling. Inactive behaviors included sleeping and awake inactive. Postures were used for this behavioral analysis; piglets that were standing or sitting were scored as active and lying piglets were scored as inactive. Sitting was scored in the active category because most piglets exhibited this posture when rump scratching or suckling and these were considered active behaviors. Pain behaviors included trembling, stiffness, spasms, tail wagging, and rump scratching [2].

#### Piglet grimace scale and scoring

Still images of piglet faces were taken from the first 30 min of every hour of video data by an individual blinded as to time point and animal treatment using the Everio MediaBrowser 4 program (Pixela Corporation, Osaka, Japan). Whenever a piglet face was in view, the video was paused, and the still image was captured (excluding times when piglets were lying with their head down or sleeping). Taking at least one facial image of each piglet per time point in this study was attempted. A total of 511 images were captured (Table 3). The images were uploaded to Photoshop (Adobe Systems Incorporated, San Jose, CA) prior to scoring to blur the symbol marked on each piglet’s forehead. This was to ensure that those scoring the faces were blinded as to treatment. Faces were then randomized into files using a random number generator (random.org).

**Table 3:** Total number of piglet faces captured for Piglet Grimace Scale scoring

Time point (h)	Treatment				Total
	0.04 mg/kg BUP cast	0.04 mg/kg BUP uncast	Saline	Sham	
pre	17	14	17	9	57
0	18	17	15	11	61
1	12	21	3	10	46
2	18	19	11	6	54
3	23	12	7	8	50
4	12	15	7	5	39
5	19	19	10	7	55
6	14	15	10	8	47
7	12	13	9	2	36
24	18	21	15	12	66
Total	163	166	104	78	511

**Fig. 8:**
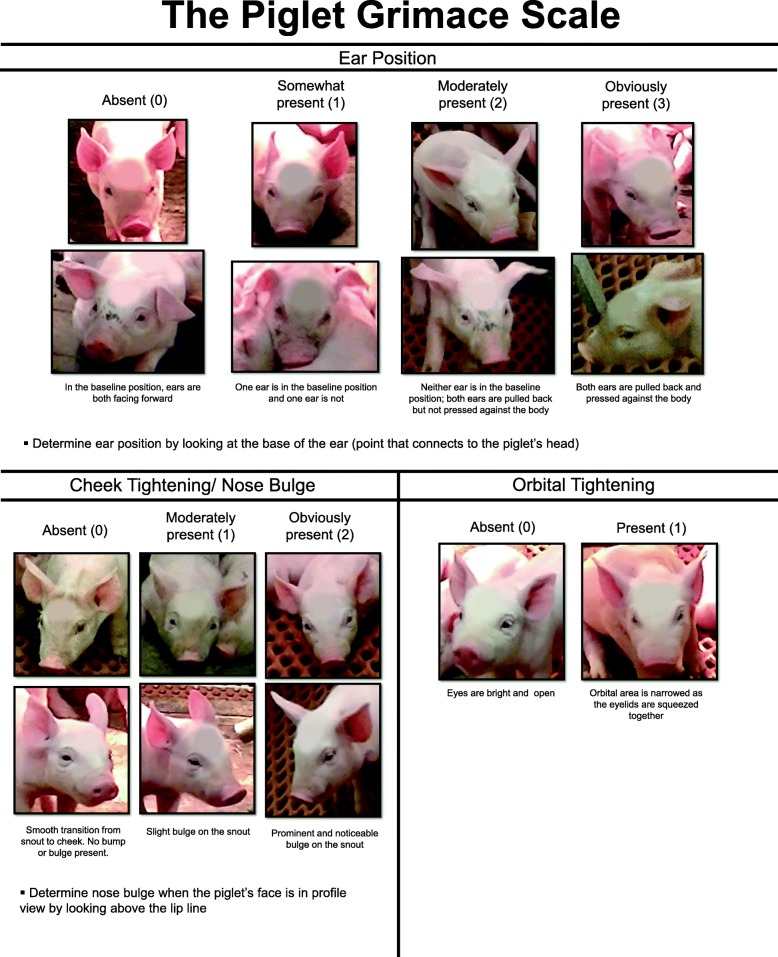
The Piglet Grimace Scale (PGS) scores ear position, cheek tightening/nose bulge and orbital tightening. The maximum score is 6

Four individuals with extensive animal experience were trained to use the Piglet Grimace Scale (Fig. 8) in a 30 min interactive training session prior to scoring study faces. If an image could not be scored reliably, those scoring were instructed to exclude it (3 images were removed in total because of poor image quality). The PGS score for each image was calculated by summing the scores given to each of the facial action units (ear position, cheek tightening/nose bulge and orbital tightening). If more than one image was pulled from the same piglet within one time point, the PGS scores were averaged across images prior to analysis, to prevent issues with pseudo-replication. The final PGS score of each piglet per time point was calculated as a mean of the scores from the four individuals.

#### Vocalizations

Vocalizations of each piglet were collected at three points during the study, at initial handling when they were marked with a symbol (marking), when they received their treatment injection (injection) and when they were surgically castrated (incision and castration). A video camera on a tripod was placed as close to the focal piglet’s face as possible and recorded each procedure. Vocalizations from the recorded videos were analyzed using Raven Pro 1.5 (Cornell Lab of Ornithology, Ithaca, NY) by two individuals who were blinded as to procedure and piglet treatment. From the spectrograms, maximum frequency (Hz), maximum amplitude (μ), maximum power (dB) and energy (dB) of each call was determined [43, 54].

#### Data and statistical analysis

The total duration of behaviors was converted into a proportion of time that piglets spent demonstrating each behavior prior to analysis to account for periods of time when piglets were out of view and unable to be scored. Normality was evaluated using the univariate procedure in SAS (Statistical Analysis System 9.4, SAS Institute Inc., NC). Data was analyzed with a GLIMMIX procedure with a beta distribution, including time, treatment, litter, and the time x treatment interaction. Litter was included as a random effect and time was a repeated measure with piglet as the experimental unit. Post hoc tests were conducted on significant factors using the Tukey-Kramer adjustment. Statistical significance was set at *P* < 0.05.

The PGS scores were analyzed using a mixed model procedure, including litter, time, treatment, and time x treatment interaction. Litter was included as a random effect, time was a repeated measure, and piglet was the experimental unit. A post-hoc Tukey’s test was conducted for significant outcomes.

Vocalization data was analyzed using a mixed procedure, including litter, treatment, and procedure in the model. Litter was included as a random effect and piglet was the experimental unit. Significant outcomes were further analyzed using a post-hoc Tukey’s test. Behavior, PGS and vocalization data were used to assess the effectiveness of buprenorphine treatment in reducing surgical castration pain.

To determine if piglet weight was balanced across treatment groups, post-hoc analysis was performed using a GLM procedure, including litter and treatment in the model.

## Abbreviations

BUP: Buprenorphine
BW: Body weight
IM: Intramuscular
NSAID: Nonsteroidal anti-inflammatory drug
PGS: Piglet Grimace Scale
